# Is It Really Feasible to Use Budesonide–Formoterol as Needed for Mild Persistent Asthma? A Systematic Review and Meta-Analysis

**DOI:** 10.3389/fphar.2021.644629

**Published:** 2021-06-04

**Authors:** Xiang Tong, Tao Liu, Zhenzhen Li, Sitong Liu, Hong Fan

**Affiliations:** ^1^ Department of Respiratory and Critical Care Medicine, West China Hospital/West China School of Medicine, Sichuan University, Chengdu, China; ^2^ Health Management Center, West China Hospital/West China School of Medicine, Sichuan University, Chengdu, China

**Keywords:** mild persistent asthma, budesonide–formoterol, used as needed, exacerbation, pulmonary function, meta-analysis

## Abstract

**Background:** Previous studies suggest that inhaled budesonide-formoterol used as needed could effectively reduce the severe exacerbation of mild persistent asthma. However, there are some differences between these studies, so we conducted a meta-analysis.

**Methods:** We searched PubMed, Ovid MEDLINE, Cochrane Library and several web search engines to screen the literature until March 25, 2020 and used risk ratios (RR), odds ratios, hazard ratios (HR) and weighted mean differences with 95% confidence intervals (CI) to evaluate the pooled effects. Adolescent/adult patients with mild persistent asthma who used budesonide–formoterol as needed were included in this study. The primary outcome was to investigate the superiority of budesonide–formoterol as needed in reducing severe exacerbations in patients with mild persistent asthma. STATA 12.0 software was used for statistical analysis.

**Results:** Across all 4 articles, 4,023 patients used budesonide–formoterol as needed (budesonide–formoterol group), 4,042 patients used budesonide maintenance plus short-acting β_2_-agonist (SABA) as needed (budesonide group), and 1,500 patients used SABA as needed (SABA group). The results showed that the incidence of severe exacerbations and the time to first severe exacerbation in the budesonide–formoterol group were significantly different from those for the SABA group (RR = 0.46, 95% CI = 0.36–0.59, *p* < 0.001; HR = 0.43, 95% CI = 0.33–0.56, *p* < 0.001; respectively), but there was no difference between the budesonide–formoterol group and budesonide group (RR = 0.86, 95% CI = 0.62–1.04, *p* = 0.093; HR = 0.77, 95% CI = 0.57–1.03, *p* = 0.079; respectively). There were statistically significant differences in the forced expiratory volume in 1 second and in the responses to the Asthma Control Questionnaire-5 between the budesonide-formoterol group and the SABA group, but the differences were not clinically significant. In addition, the daily dose of budesonide in the budesonide–formoterol group was significantly lower than that in the budesonide group, and there was no difference in the incidence of adverse events among the three groups.

**Conclusion:** In summary, budesonide–formoterol used as needed may reduce severe exacerbation in adolescent/adult patients with mild persistent asthma.

## Introduction

Asthma is a heterogeneous lung disease and an important global public health problem affecting all age groups that is usually characterized by chronic airway inflammation (Global Initiative for Asthmam, 2019). Among asthma patients, the proportion with mild asthma should be 50–70% (Dusser et al., 2007). In general, patients with mild asthma have few or intermittent symptoms, so their treatment adherence is poor, especially with regard to the regular use of low-dose inhaled corticosteroids (ICS) as maintenance treatment (Rand et al., 2007). However, it should be noted that they still present with airway inflammation and have an increased risk of exacerbation and asthma-related death due to inadequate use of inhaled glucocorticoids (Fuhlbrigge et al., 2002; Barnes and Ulrik, 2015). For example, a study from the United States found that 3.6% of patients with mild asthma were hospitalized due to deterioration of their condition; 16.1% of patients with intermittent asthma and 28.4% of patients with mild asthma needed emergency treatment in the previous year (Fuhlbrigge et al., 2002). Short-acting β_2_-agonist (SABA) is widely used to relieve the symptoms of mild asthma patients, and overuse is common (Belhassen et al., 2016). Previous studies have shown that the use of SABA may increase the risk of exacerbation, disease progression and death in patients with asthma (Suissa et al., 1994; Stanford et al., 2012). Therefore, whether there is a better alternative strategy for the treatment of mild persistent asthma has been the subject of concern in recent years.

Several recent studies have found that a combination of inhaled corticosteroids and formoterol [a fast- and long-acting β-agonist (LABA)] used as needed is an alternative treatment for mild persistent asthma. In the SYmbicort Given “as needed” in Mild Asthma (SYGMA) studies, compared with SABA used as needed, ICS-LABA can effectively reduce the risk of exacerbation and improve symptoms, and it was not inferior to low-dose ICS maintenance therapy in patients with mild asthma (Bateman et al., 2018; O’Byrne et al., 2018). Two studies published since then have yielded somewhat similar results (Beasley et al., 2019; Hardy et al., 2019). In these studies, it is worth noting that, although some of the results in these studies are significantly different, they are not close to the minimum absolute difference required for clinical significance. Additionally, there are still some differences between the results of these studies. The latest Global Initiative for Asthma (GINA) guidelines recommend that patients with mild asthma should follow as-needed treatment with ICS-LABA instead of SABA alone (Global Initiative for Asthmam, 2019), but there is still lack of systematic evidence, and no meta-analysis of the recently published literature has been conducted. Therefore, we conducted this study.

## Methods

### Literature Search

We performed a systematic literature search for studies in which budesonide–formoterol was used as needed to treat mild persistent asthma in PubMed, Ovid MEDLINE and Cochrane Library, with the last updated search conducted on March 25, 2020. The key search words were as follows: “budesonide–formoterol” OR “inhaled corticosteroids” OR “ICS-LABA” and “mild asthma” and “as needed.” There was no language restriction. Moreover, we conducted a web-based search (such as Google Scholar, Baidu Scholar and ClinicalTrials.gov) using similar terms. All analyses in the present meta-analysis were based on previously published studies; thus, no ethical approval was required.

### Study Selection

The inclusion criteria were defined as follows: 1) randomized-controlled studies; 2) studies evaluating the efficacy of treatment with budesonide–formoterol used as needed in mild persistent asthma patients; and 3) primary studies providing available data for calculating risk ratios (RR), hazard ratios (HR) and weighted mean difference (WMD) with 95% confidence intervals (CI).

### Data Extraction

The detailed information and data from each primary study were independently extracted by two authors (Xiang Tong and Tao Liu) by using a predesigned data extraction Microsoft® Excel® form. If there was any doubt or disagreement, the third author (Zhenzhen Li) further reviewed these articles. The extractions included the following: first author, year of publication, designed method, sample sizes, age of participants, intervention drugs and doses, number of severe exacerbations, annualized severe exacerbation rate, the HR of the first time to severe exacerbation between groups, mean differences in the Asthma Control Questionnaire-5 (ACQ-5) score and forced expiratory volume in 1 second (FEV_1_), and adverse events.

### Statistical Methods

In the current study, all data analyses were performed using STATA 12.0 software. The HR, RR, or WMD with 95% CI was used to investigate the efficacy of budesonide–formoterol used as needed in patients with mild persistent asthma. Based on the control group, the studies were divided into the following two categories: The first included patients who inhaled budesonide twice per day regularly and used SABA as needed, and the second included patients who only inhaled SABA as needed. Random effects models are more conservative when they are used to pool data, and they lead to a lower type I error rates and wider CI of effects in comparison to fixed effects models (Borenstein et al., 2007). As such, in the present meta-analysis, we used random effects modeling to combine the data. The between-study heterogeneity was investigated by using the chi-square (*χ* ^2^)-based Q-test and I-square (I ^2^) statistics test. An I^2^ value of >50% or a *p* value of <0.10 suggests statistically significant heterogeneity.

## Results

### Study Characteristics

**FIGURE 1:**
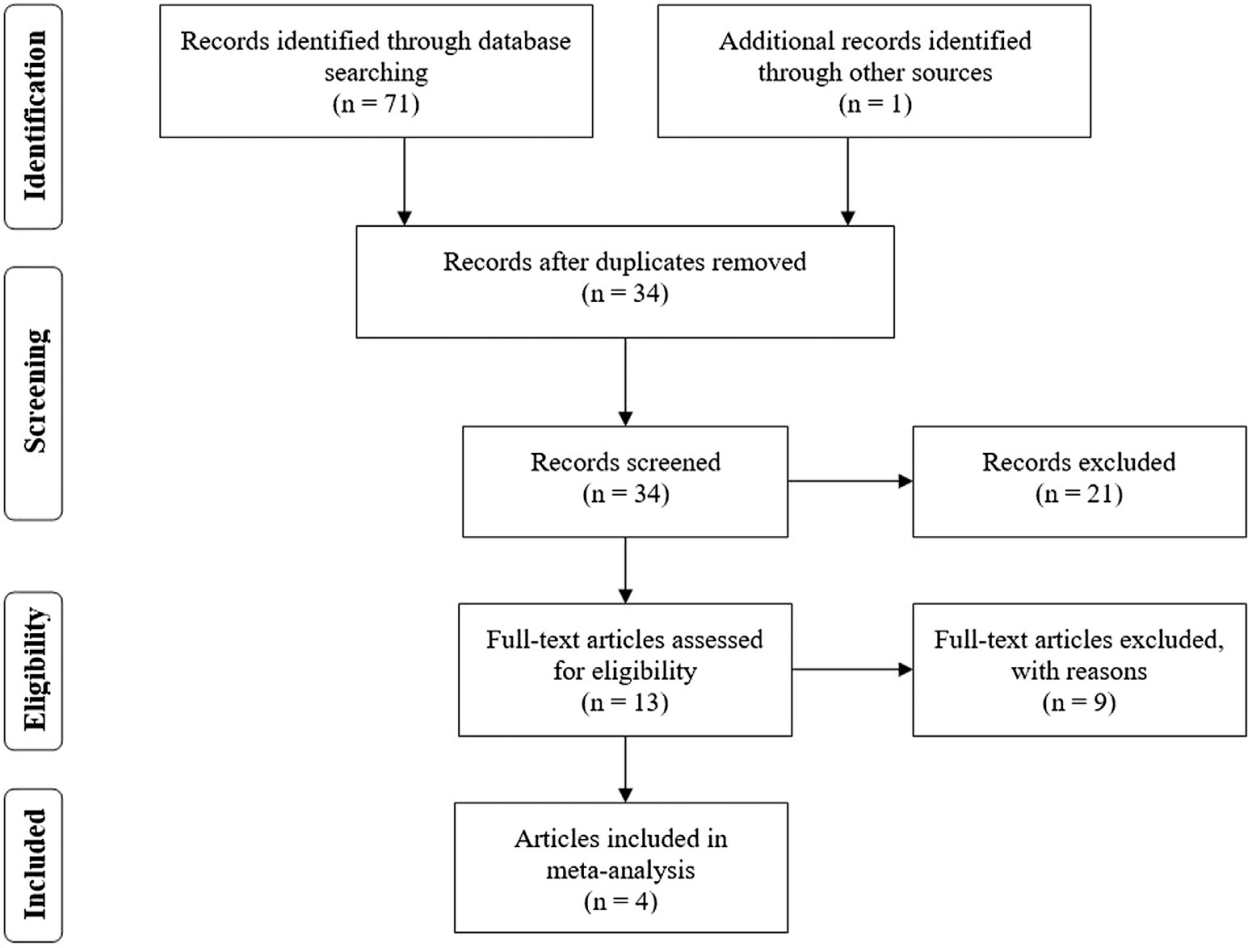
The flow diagram of included and excluded studies.

Based on the search of PubMed, Ovid MEDLINE, Cochrane Library and several web search engines, a total of 72 articles initially conformed to the search strategy. As shown in the flow chart (Figure 1), 38 articles were eliminated on account of being duplicated across databases. After we carefully read the titles and abstracts, 21 articles were excluded because they did not meet the inclusion criteria. Therefore, the remaining 13 articles underwent a full-view screen. Two articles were deleted because they described research design schemes rather than data. Three articles were excluded because they were reviews. Two articles were excluded because they were commentaries or editorials. One study mainly explored the burden of medical costs, and the other study was inconsistent with the purpose of our study, so those two articles were not included. A final total of four articles (Bateman et al., 2018; O’Byrne et al., 2018; Beasley et al., 2019; Hardy et al., 2019), consisting of 4,023 patients who used budesonide–formoterol as needed (budesonide–formoterol group), 4,042 patients who used budesonide for maintenance plus SABA as needed (budesonide group) and 1,500 patients who used SABA as needed (SABA group), were included in the current meta-analysis. In one of these studies, some patients with moderate asthma were included, but the exact proportion is unknown (Hardy et al., 2019). Table 1 summarizes the characteristics of the patients included in this study.

**TABLE 1:** Characteristics of included studies.

Author (year)	Intervention comparisons	Sample sizes	Duration (weeks)	Age	No. of severe exacerbation	Annual rate	First time to severe exacerbation		ACQ-5		FEV1		Inhaled glucocorticoid dose	No. of adverse event	No. of severe adverse event
						RR	HR								
						1 vs 2	1 vs 3	1 vs 2	1 vs 3	1 vs. 2	1 vs 3	1 vs 2			
O’Byrne et al. (2018)	Budesonide/formoterol 200/6 μg PRN (group 1)	1,277	52	39.8 ±16.9	71	0.83 (0.59–1.16)	0.44 (0.33–0.58)	0.90 (0.65–1.24)	−0.15 (−0.20 to −0.11)	0.15 (0.10–0.20)	53.8 (29.1–78.5)	−54.3 (−78.8 to −29.8)	92.9 ± 102.2	485	38
	Budesonide 200 μg BID + terbutaline 0.5 mg PRN (group 2)	1,282		39.0 ± 16.7	78								314.9 ± 89.2	512	37
	Terbutaline 0.5 mg PRN (group 3)	1,277		40.0 ± 16.3	152									545	50
Bateman et al. (2018)	Budesonide/formoterol 200/6 μg PRN (group 1)	2,089	52	41.3 ± 16.8	177			0.96 (0.78–1.17)		0.11 (0.07–0.15)		−32.6 (−53.7 to −11.4)	103.5 ± 109.3	887	66
	Budesonide 200 μg BID + terbutaline 0.5 mg PRN (group 2)	2087		40.7 ± 17.1	184	0.97 (0.78–1.20)							250.6 ± 117.6	919	73
Beasley et al. (2019)	Budesonide/formoterol 200/6 μg PRN (group 1)	220	52	36.0 ± 14.1	9		0.38 (0.18–0.82)	0.41 (0.19–0.90)	−0.15 (−0.24 to −0.06)	0.14 (0.05–0.23)	30 (−6 to 70)	4 (−0.30 to 40) L	107 ± 109	174	13
	Budesonide 200 μg BID + albuterol 0.1 mg PRN (group 2)	225		34.9 ± 14.3	21								222 ± 113	190	7
	Albuterol 0.1 mg PRN (group 3)	223		35.8 ± 14.0	23									185	6
Hardy et al. (2019)	Budesonide/formoterol 200/6 μg PRN (group 1)	437	52	43.3 ± 15.2	37			0.60 (0.40–0.91)		0.06 (–0.005–0.12)		6 L (–26 to 40)	176.0 ± 143.0	385	28
	Budesonide 200 μg BID + terbutaline 0.5 mg PRN (group 2)	448		42.8 ± 16.7	59								302.5 ± 84.8	371	16

ACQ-5, asthma control questionnaire; HR, hazard ratio; FEV1, forced expiratory volume in 1 second; RR, relative ratio.

### Severe Exacerbations

**FIGURE 2:**
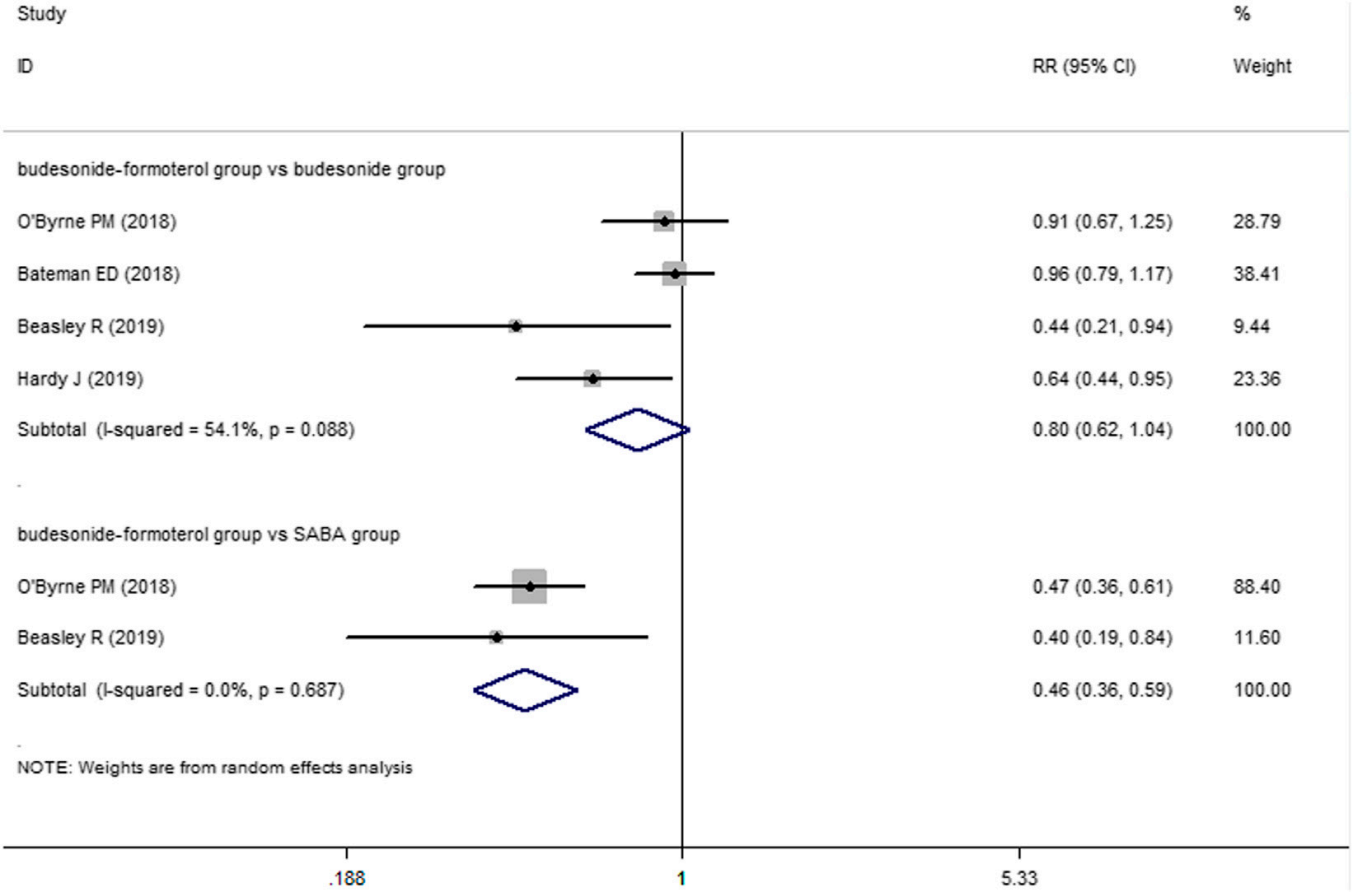
Comparison of the difference of severe exacerbations patients in the three treatment groups.

**FIGURE 3:**
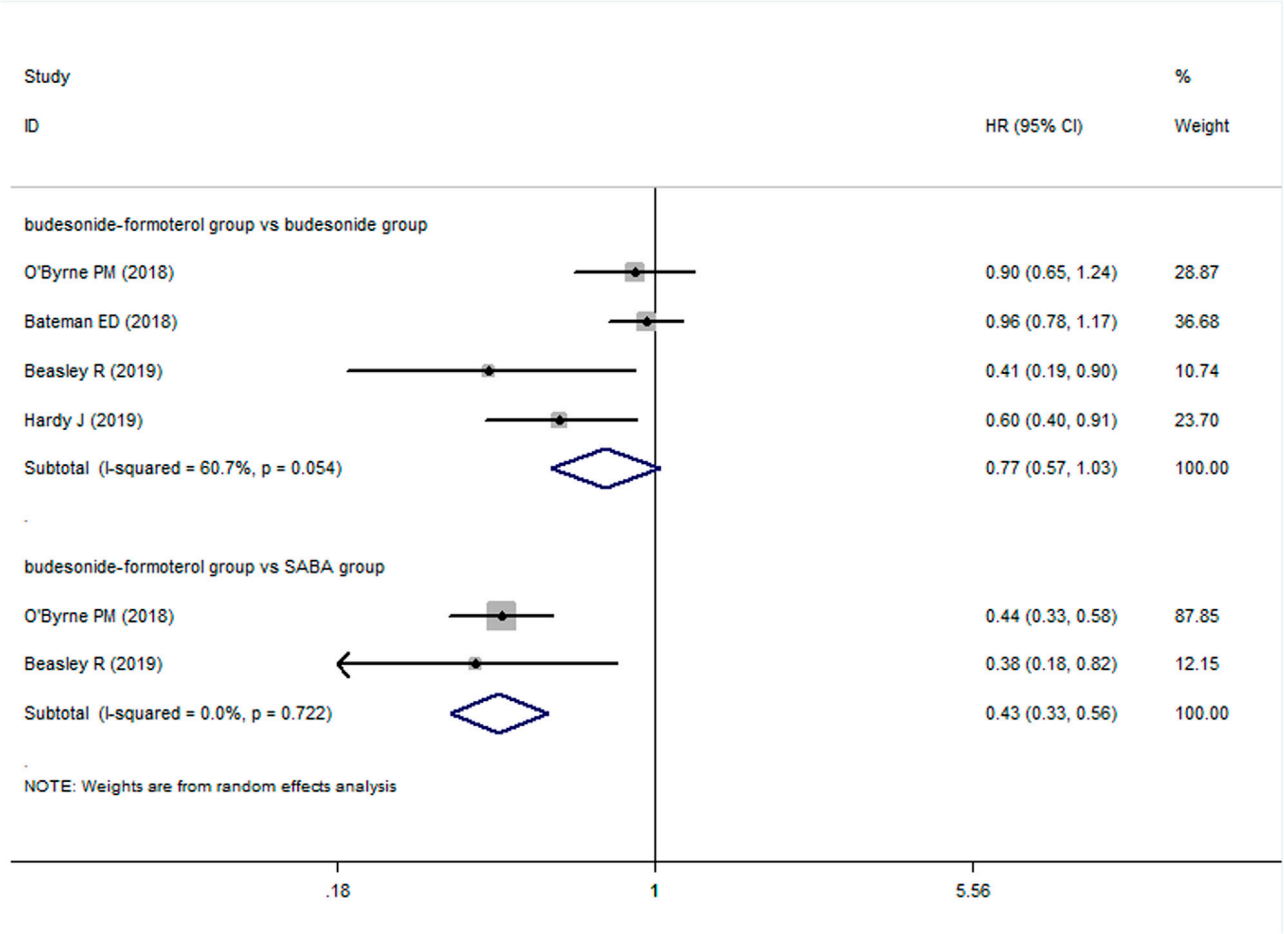
Comparison of the difference of time to first severe exacerbation in the three treatment groups.

For the number of patients with severe exacerbations, two included studies compared the budesonide–formoterol group and the SABA group, and four studies provided data on the budesonide–formoterol group and the budesonide group. The results showed that there was a significant difference between the budesonide–formoterol group and the SABA group (RR = 0.46, 95% CI = 0.36–0.59, *p* < 0.001), but there was no significant difference between the budesonide–formoterol group and the budesonide group (RR = 0.86, 95% CI = 0.62–1.04, *p* = 0.093) (Figure 2). In addition, the annual rates of severe exacerbations were compared between the budesonide–formoterol group and the budesonide group in three studies. The results showed that there was no significant difference between the budesonide–formoterol group and the budesonide group (RR = 0.86, 95% CI = 0.71–1.04, *p* = 0.127). For time to first severe exacerbation, the meta-analysis found that there was a significant difference between the budesonide–formoterol group and the SABA group (HR = 0.43, 95% CI = 0.33–0.56, *p* < 0.001), but there was no significant difference between the budesonide–formoterol group and the budesonide group (HR = 0.77, 95% CI = 0.57–1.03, *p* = 0.079) (Figure 3). Significant heterogeneity was detected when comparing the number of patients with severe exacerbation and time to first severe exacerbation between the budesonide formoterol group and the budesonide group (I^2^ = 54.1%, I^2^ = 60.7%, respectively).

### Forced Expiratory Volume in 1 Second and Asthma Control Questionnaire-5

**FIGURE 4:**
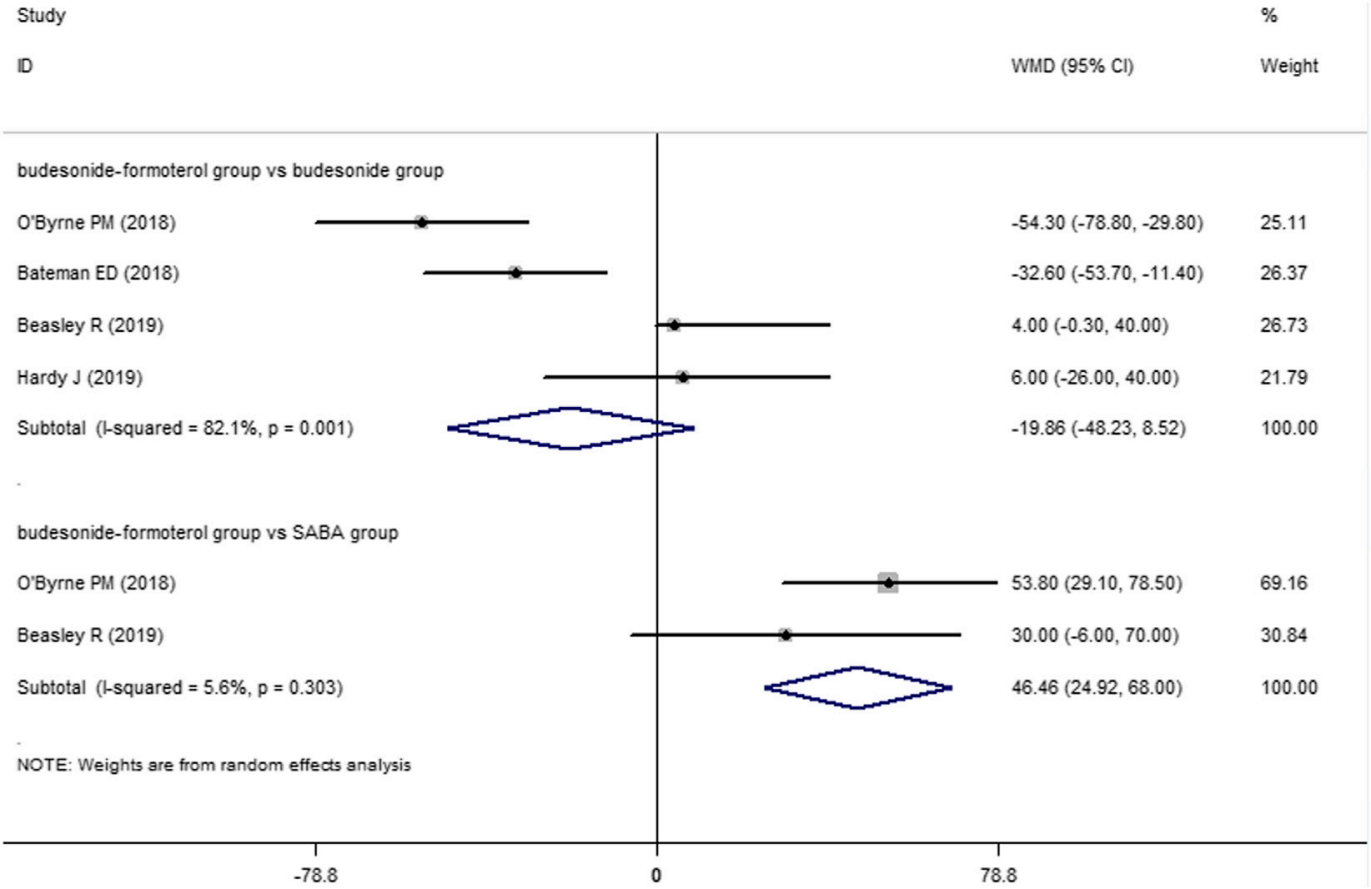
Comparison of the difference of change in the FEV1 in the three treatment groups.

**FIGURE 5:**
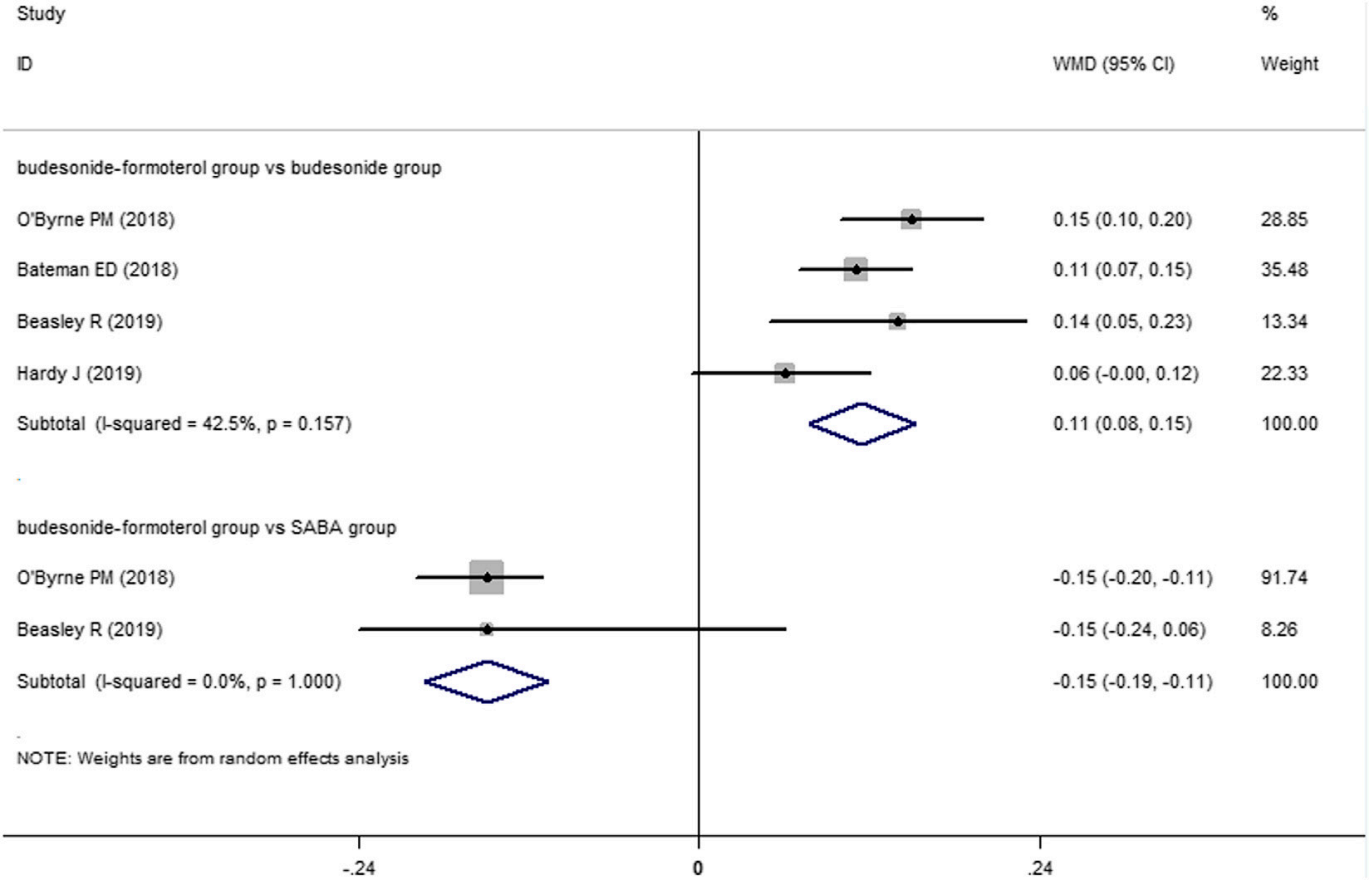
Comparison of the difference of change in the ACQ-5 in the three treatment groups.

Two of the four included studies reported a comparison of the change in the FEV1 between the budesonide–formoterol group and the SABA group, and all four studies provided comparisons of the change in the FEV1 between the budesonide–formoterol group and the budesonide group. The meta-analysis results showed that there was a significant difference in the change in the FEV1 between the budesonide–formoterol group and the SABA group (WMD = 46.46, 95% CI = 24.92–68.0, *p* < 0.001), but there was no significant difference in the change in the FEV1 between the budesonide–formoterol group and the budesonide group (WMD = −19.86, 95% CI = −48.23 to 8.52, *p* = 0.17) (Figure 4). For ACQ-5 scores, the results showed that there were significant differences in the change in the ACQ-5 scores between the budesonide–formoterol group and the SABA group as well as between the budesonide-formoterol group and the budesonide group [WMD = −015, 95% CI = −0.19 to (−0.11), *p* < 0.001; WMD = 0.11, 95% CI = 0.08–0.15, *p* < 0.001; respectively] (Figure 5). Significant heterogeneity was detected when comparing the changes in the FEV1 and ACQ-5 scores between the budesonide formoterol group and the budesonide group (I^2^ = 82.1%, I^2^ = 42.5%, respectively).

### Inhaled Glucocorticoid Dose and Adverse Events

The mean daily inhaled glucocorticoid dose was compared between the budesonide–formoterol group and the budesonide group in all four studies. The results of the meta-analysis showed that there was a significant difference between the two groups [WMD = −154.0, 95% CI = −206.87 to (−101.14), *p* < 0.001]. In addition, in the comparison of adverse events among the groups, there was no significant difference between the budesonide–formoterol group and the SABA group or between the budesonide-formoterol group and the budesonide group (RR = 0.92, 95% CI = 0.85–1.0, *p* = 0.053; RR = 0.98, 95% CI = 0.92–1.05, *p* = 0.587; respectively). Similarly, there was no significant difference in the incidence of serious adverse events between the three groups (RR = 1.22, 95% CI = 0.44–3.38, *p* = 0.699; RR = 1.18, 95% CI = 0.84–1.66, *p* = 0.344; respectively). We also further explored whether there was any difference in rates of upper respiratory tract infection among the three groups and found no difference between the three groups (RR = 0.91, 95% CI = 0.79–1.05, *p* = 0.202; RR = 0.96, 95% CI = 0.79–1.18, *p* = 0.68; respectively). Significant heterogeneity was also detected when comparing the daily inhaled glucocorticoid dose and adverse events between the budesonide formoterol group and the budesonide group (I^2^ = 98.8, I^2^ = 67.7%, respectively).

## Discussion

According to the results of this meta-analysis, compared with SABA used as needed, budesonide–formoterol used as needed can effectively reduce the risk of severe exacerbation of mild persistent asthma: that is, budesonide-formoterol used as needed resulted in a 54% lower rate of severe exacerbation (defined as worsening asthma leading to the prescription of systemic glucocorticoid treatment for ≥3 days, or hospitalization or an emergency department visit leading to systemic glucocorticoid treatment (Patel et al., 2013). In addition, budesonide–formoterol used as needed can effectively prolong the time to first severe exacerbation of mild persistent asthma patients in comparison to SABA used as needed. There was no difference in the risk of severe exacerbation or the time to first severe exacerbation between the budesonide–formoterol group and the budesonide group. Moreover, in SYGMA studies, the rate of adherence was good, and there was no significant difference between the groups (Bateman et al., 2018; O’Byrne et al., 2018). Interestingly, a recent study has shown that budesonide–formoterol used as needed in patients with mild asthma can effectively reduce medical costs (Elsisi et al., 2019). Thus, these findings may result in a series of favorable effects on mild persistent asthma patients; that is, if they are treated with budesonide–formoterol used as needed, patients’ medical compliance can be increased, and the cost burden and mortality can be decreased (Elsisi et al., 2019).

For the treatment and management of asthma, lung function improvement and symptom control are additional concerns. Three previous studies found that the ACQ-5 scores of patients in the budesonide–formoterol group were higher than those of the budesonide group (Bateman et al., 2018; O’Byrne et al., 2018; Beasley et al., 2019), but the latest study completed by Hardy et al. did not find similar results (Hardy, et al., 2019). The results of this meta-analysis showed that the ACQ-5 scores in the budesonide–formoterol group were higher than those of the budesonide group but lower than those of the SBAB group [WMD = −015, 95% CI = −0.19 to (−0.11), *p* < 0.001; WMD = 0.11, 95% CI = 0.08–0.15, *p* < 0.001; respectively]. It should be noted that these differences in ACQ-5 scores did not reach the minimum threshold of clinical significance. For FEV1, the meta-analysis showed that the budesonide–formoterol group experienced significantly greater improvement than the SABA group (WMD = 46.46, 95% CI = 24.92–68.0, *p* < 0.001), but there was no difference between the budesonide–formoterol group and the budesonide group (WMD = −19.86, 95% CI = −48.23 to 8.52, *p* = 0.17). Similarly, the absolute value of FEV1 improvement was limited for clinical value evaluation. There are differences between the results of our meta-analysis and the results of previous SYGMA studies (Bateman et al., 2018; O’Byrne et al., 2018), which may be related to the slightly different design methods and inclusion criteria used in the two later studies published in 2019. More studies may be needed in the future to assess whether budesonide–formoterol used as needed by mild persistent asthma patients can improve lung function and control symptoms.

Corticosteroid dose exposure is also a concern in asthma treatment and management. Previous studies have found that long-term inhaled corticosteroid may lead to a series of adverse events, although many findings may still be controversial. For example, long-term use of inhaled corticosteroid in asthma patients could increase risk of upper respiratory tract infection [odds ratio (OR) = 1.24, 95% CI = 1.08–1.42] (Yang et al., 2018), pneumonia (OR = 1.38, 95% CI = 1.36–1.41) (Kim et al., 2019), gastrointestinal events (HR = 1.26, 95% CI = 1.02–1.56) (Hansen et al., 2008) and so on. Differences in the daily dose of inhaled corticosteroid between the groups was reported in four published articles (Bateman et al., 2018; O’Byrne et al., 2018; Beasley et al., 2019; Hardy et al., 2019). This meta-analysis showed that the daily dose of inhaled corticosteroid in the budesonide–formoterol group was significantly lower than that in the budesonide group [WMD = −154.0, 95% CI = −206.87 to (−101.14), *p* < 0.001]. However, our meta-analysis did not find any significant difference in the incidence of adverse events, including upper respiratory tract infection, among the groups. It is worth noting that the treatment of asthma is a long-term process, and the risk of respiratory infection still exists and may be closely related to the age of the patients, the environment and occupational exposure, complications, the duration of inhaled drug use and the correct use of inhaled treatment measures (D’Amato et al., 2014; Job et al., 2016).

To summarize, our meta-analysis found that budesonide–formoterol used as needed for mild persistent asthma may significantly reduce severe exacerbations and prolong the time to first severe exacerbation compared with SABA used as needed, but there may be no clinically significant difference in symptom control and lung function improvement, which needs to be confirmed by further research. Thus, some concerns are should be considered. For instance, Domingo et al. contemplated their review whether reducing severe exacerbations or controlling symptoms was more important for asthma patients (Domingo et al., 2019). They arrived at no definite conclusion. In addition, long-term uncontrolled inflammation is one of the risk factors recognized for airway remodeling (Cohn et al., 2004). Although two studies found that the use of budesonide–formoterol used as needed can reduce the level of FeNO, which reflects that it may be beneficial to airway inflammation, the FeNO level was higher than that in the budesonide maintenance group (Novel START study: ratio of geometric means, 1.13; 95% CI, 1.02 to 1.25; PRACTICAL study: ratio of geometric means 1.13, 95% CI 1.07–1.21) (Beasley et al., 2019; Hardy et al., 2019). Due to the complexity of the pathogenesis of airway remodeling, whether budesonide–formoterol used as needed can fully improve airway inflammation in comparison to budesonide maintenance will have implications for future airway remodeling; thus, it deserves further attention. Moreover, although some patients with moderate asthma were included (Hardy et al., 2019), there were not enough patients and evidence to show that use of budesonide–formoterol as needed is definitely beneficial for patients with moderate asthma.

Although several reviews had evaluated the efficacy of budesonide–formoterol used as needed in patients with mild asthma (Bianco et al., 2020; Cloutier et al., 2020; Papi et al., 2021), it was still not comprehensive. In our meta-analysis, we not only showed that inhaled budesonide-formoterol used as needed may reduce severe exacerbations in patients with mild persistent asthma, but also emphasized that the evidence of budesonide-formoterol used as needed for improving clinically significant difference of symptoms and lung function may be insufficient, which needs to be further confirmed in the future. Different from other reviews (Bianco et al., 2020; Cloutier et al., 2020; Papi et al., 2021), we conducted quantitative analysis of published data of previous literatures, which is helpful for physicians to understand more vividly. It’s worth noting that there was a high degree of heterogeneity when comparing multiple outcomes (exacerbation, FEV1, inhaled glucocorticoid dose and adverse events) between the budesonide group and the budesonide-formoterol group in the present meta-analysis. This heterogeneity may be due to differences in study design, implementation strategy, patient baselines, patient composition, and sample size. Additionally, when we pooled the data, some of the results reflected the direct effects provided by the original research, so there may have been bias. Moreover, due to the limited data, some of the outcomes were not fully combined.

## Conclusion

In summary, compared with SABA treatment used as needed, budesonide–formoterol used as needed may reduce severe exacerbations in adolescent/adult patients with mild persistent asthma; however, whether budesonide–formoterol can improve lung function and symptom control requires further verification. Additionally, it was difficult to confirm whether budesonide formoterol used as needed was not inferior to budesonide maintenance treatment for patients with mild persistent asthma at this stage.

## Data Availability

The original contributions presented in the study are included in the article/Supplementary Material, further inquiries can be directed to the corresponding author.
